# Enhancing Alzheimer’s disease diagnosis in primary care through targeted screening of people at risk of developing dementia – study design and feasibility assessment of the PREDEM primary care study

**DOI:** 10.1186/s13195-026-02162-w

**Published:** 2026-09-14

**Authors:** Sandar Aye, Signe Åhrberg, Axel C Carlsson, Jan Hasselström, Ibrahim Alshamani, Sara Wallen, Wobbie van den Hurk, Lena Lund, Alenka Jejcic, Sarah Thomas Broome, Adrià Duran Sidera, Lars Tjernberg, Linus Jönsson, Sophia Schedin-Weiss

**Affiliations:** 1 Division of Neurogeriatrics, Department of Neurobiology, Care Sciences and Society, Karolinska Institutet, BioClinicum J9:20, Visionsgatan 4, Solna, 17164 Sweden; 2 Division of Family Medicine and Primary Care, Department of Neurobiology, Care Sciences and Society, Karolinska Institutet, Alfred Nobels allé 23, Huddinge, A4 141 83 Sweden; 3 Academic Primary Health Care Centre, Region Stockholm, Stockholm, Sweden; 4 Mindmore AB, Stockholm, Sweden; 5 Department of Medicine and Health Sciences, Linköping University, Linköping, Sweden; 6 Clinical Chemistry, Medical Diagnostics Karolinska, Karolinska University Hospital, Stockholm, Sweden

**Keywords:** Alzheimer’s disease, Dementia, Targeted screening, Primary care, Digital cognitive test, Blood-based biomarkers, Feasibility study

## Abstract

**Background:**

Timely detection of cognitive impairment due to Alzheimer’s disease (AD) is increasingly important as disease modifying therapies become available. Current diagnostic pathways in primary health care (PHC) rely mainly on clinical assessment and brief cognitive screening tools, which lack sensitivity for early impairment. Blood-based biomarkers (BBMs) show promising diagnostic potential in research settings. Targeted screening of individuals with cognitive symptoms and dementia risk factors using sensitive cognitive tests and reliable biomarkers may improve early AD detection in PHC. The PREDEM primary care study evaluates a targeted screening strategy combining a multidomain digital cognitive test battery and BBMs in individuals having elevated risk of dementia. This pilot study assessed the feasibility of recruitment, digital cognitive testing, biomarker sampling, and data collection.

**Methods:**

This descriptive pilot study was conducted at two PHC centres in Stockholm (April–October 2024). Eligible individuals were aged ≥ 65 years with hypertension or type 2 diabetes and self-reported memory complaints. Participants completed a screening question, followed by a demographic questionnaire, a multidomain digital cognitive test battery (Mindmore), MMSE, and blood sampling for four BBMs (GFAP, NF‑L, total tau, pTau217). Feasibility metrics included recruitment yield, test completion rates, participant characteristics, cognitive performance, and biomarker distributions.

**Results:**

Of 548 identified individuals, 307 were invited and 40 were enrolled (13% participation). Among those enrolled, 37 completed all parts of the study. Digital home-based testing proved challenging: although 22 participants initially opted for home testing, only six completed it independently, and most required clinic-based support. Across cognitive tests, approximately one-third of participants scored below 1 standard deviation of normative performance, indicating detectable cognitive impairment. Non-native Swedish speakers (32%) showed significantly lower performance on verbally mediated tasks. Established cut-off values for pTau217 reflecting amyloid pathology showed pathological levels in 10–23% of participants depending on single or double cut-offs.

**Conclusions:**

This pilot study demonstrates the feasibility of the PREDEM primary care study design and data collection procedures. Targeted recruitment of older adults with cardiometabolic risk factors was able to identify a population relevant to the study aims. Key protocol refinements include removing the requirement for subjective memory complaints, conducting all cognitive testing at PHC, and optimization of cognitive test battery.

**Supplementary Information:**

The online version contains supplementary material available at https://doi.org/10.1186/s13195-026-02162-w.

## Introduction

Alzheimer’s disease (AD) is a progressive neurodegenerative disease characterized by cognitive decline, behavioural symptoms and functional impairment [1, 2]. The progression of AD follows a continuum, divided into a preclinical phase, mild cognitive impairment (MCI), mild and moderate to severe dementia [3]. The underlying pathology, involving amyloid β-peptide (Aβ) and tau depositions in the brain, is thought to begin at least 10–20 years before symptom onset [4].

In current clinical practice in several countries, the diagnostic process often begins in primary health care (PHC) when patients or family members report subjective memory complaints [5, 6]. Initial assessments typically include clinical evaluations, cognitive screening with instruments such as Mini-Mental State Examination (MMSE) or Montreal Cognitive Assessment (MoCA) along with laboratory tests and structural imaging to evaluate symptoms and exclude other causes of cognitive impairment [7]. Accordingly, dementia diagnoses in PHC are mainly based on clinical criteria. Patients with an inconclusive diagnosis are referred to specialist memory clinics for further evaluation using neuropsychological testing, brain magnetic resonance imaging (MRI), and biomarker analyses [7]. As a result, many individuals with cognitive impairments remain undiagnosed or misdiagnosed at the primary care level [8]. Even in specialist settings, AD is often diagnosed at later stages of the clinical spectrum when cognitive and functional impairments are prominent [9]. This delay in diagnosis contributes to a global socioeconomic burden of 1.7 trillion US$, largely driven by the costs of social and informal care associated with functional impairment and dependency [10].

The availability of disease-modifying therapies for AD [11, 12] highlights the inadequacy of current diagnostic approaches. Early diagnosis of cognitive impairment due to AD, set by a combination of clinical assessment and biomarker evaluation, is essential to initiate treatment that can slow disease progression [11, 12]. An early diagnosis of cognitive impairment due to other causes also facilitates management of treatable causes of cognitive impairment, lifestyle interventions, and care planning [8, 13, 14]. However, instruments commonly used in PHC, such as MMSE and MoCA, lack the sensitivity to detect subtle impairments in the early stages [15]. Thus, neuropsychological test batteries that can detect early cognitive impairment in AD, specifically in the episodic memory and executive function domains [16, 17], are critically needed in PHC.

In the context of disease-modifying therapies, detection of AD biomarkers has become increasingly important. The international working group (IWG) has long advocated for defining AD as a clinicopathological entity where diagnosis of AD requires clinical symptoms supported by biomarker evidence [18]. Despite this, biomarker confirmation is not routinely integrated into current clinical practice due to limited availability in PHC and underutilization in memory clinics [19]. The advances in blood-based biomarkers (BBMs) for AD have facilitated their use in clinical settings. These biomarkers, especially Tau protein phosphorylated at position 217 (pTau217), demonstrate promising diagnostic accuracy in research settings and in specialist care [20–22]. However, BBMs in the general population showed low positive predictive value for future AD dementia, while the negative predictive value was high [23]. Thus, further evaluation is required to optimize the use of cognitive tests and BBMs in primary care.

A key question that remains to be answered is which population(s) should undergo cognitive and BBMs testing. Universal screening of all patients visiting PHC is not recommended due to insufficient evidence on risks and benefits [24] and the increased workload for already burdened PHC physicians [25]. Targeted screening of individuals with cognitive symptoms and risk factors for developing dementia [26] would be more appropriate using highly sensitive neuropsychological test batteries to detect early cognitive impairment, and specific biomarkers to detect underlying AD pathology. PHCs are well-positioned for such screening as they are often the first point of contact for individuals with memory concerns and are responsible for triaging patients for specialist evaluation [27]. However, PHC practitioners often face time constraints that limit the use of comprehensive neuropsychological assessments [25]. Digitalization of these assessments enables self-administration and automated scoring, reducing physician involvement [28, 29]. Digitized testing also allows comparison to normative data adjusted for demographic variables such as age, sex and education, and promotes standardization of diagnostic procedures in primary care [24, 25].

The overarching aims of the PREDEM-primary care study are to:

1. evaluate the diagnostic value of combining a multidomain digital cognitive test battery with blood-based biomarkers for AD in individuals at risk of developing dementia.
2. understand the demographic and clinical characteristics of individuals reached through this targeted screening.
3. evaluate the cost-effectiveness of this approach.

A pilot study was conducted prior to full implementation to assess the feasibility of baseline participant recruitment and data collection for the PREDEM-primary care study.

## Method

### Study design

**Fig. 1:**
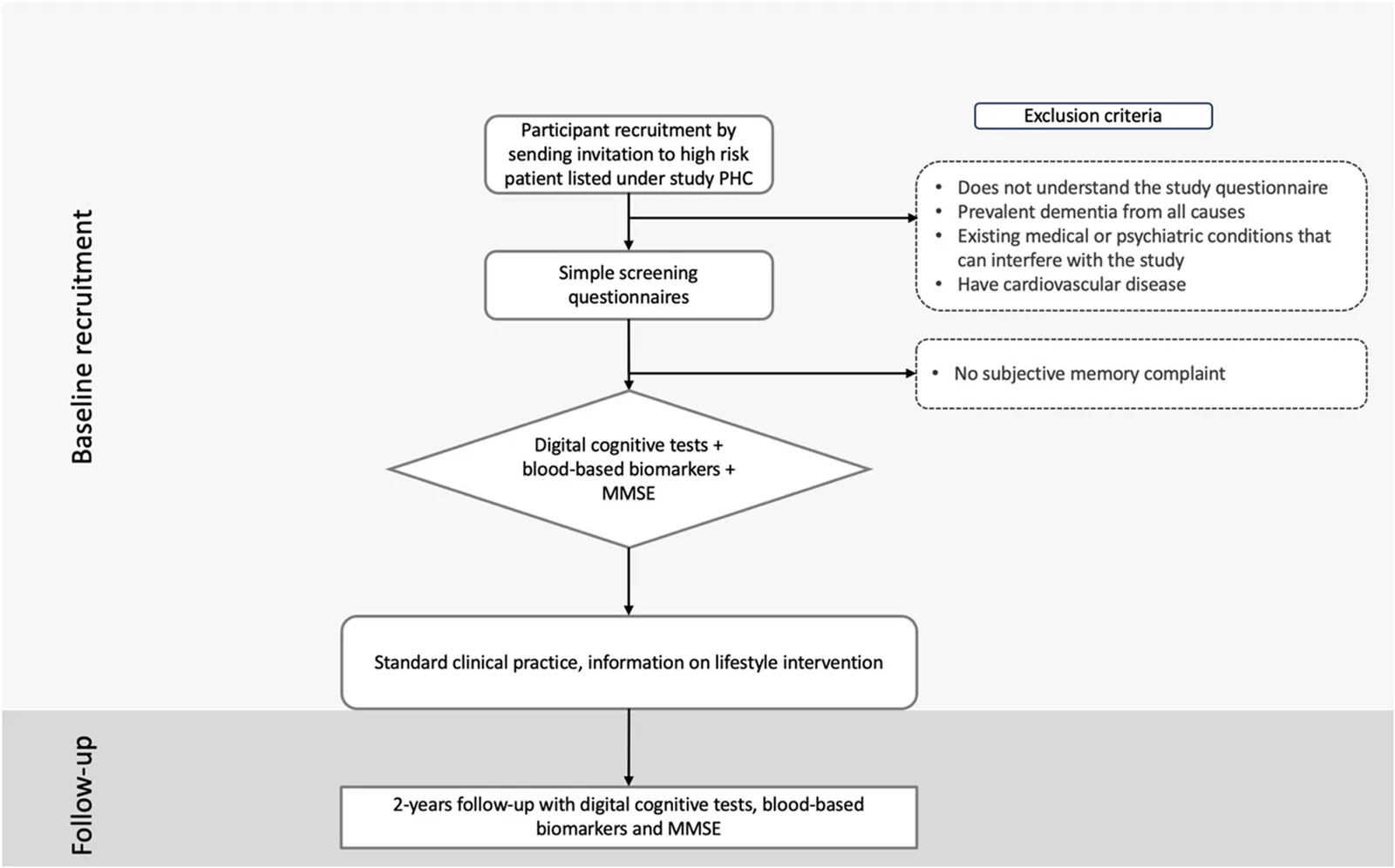
Study design of PREDEM-primary care study. Note. The pilot study assesses the participant recruitment process at baseline

This is a descriptive pilot study to test the feasibility of the baseline recruitment and workflow in the study protocol, as shown in Fig. 1. The pilot was conducted from April 2024 to October 2024 at two PHC centers in the Stockholm region.

### Study population

The study targeted individuals at risk of developing dementia who were registered at participating PHC centres in the Stockholm region. Risk was defined as being aged 65 years or older, having a diagnosis of hypertension or type 2 diabetes as recorded in PHC electronic medical records. Eligibility criteria included being registered at a participating PHC centre, aged ≥ 65 years, and responding “yes” to a screening question about memory complaints. Exclusion criteria included inability to provide informed consent or understand the study language, a history of major vascular diseases including ischemic heart diseases and stroke, presence of dementia or other neurodegenerative disorders, alcohol or drug abuse, and a history of medical or psychiatric conditions that could interfere with participation. Participants were stratified into three risk groups: (a) aged ≥ 65 years without hypertension or type 2 diabetes, (b) aged ≥ 65 years with hypertension only, and (c) aged ≥ 65 years with type 2 diabetes, with or without hypertension.

A total of 1200 study participants (400 per risk stratum) will be recruited for the full PREDEM-primary care study, assuming a twofold increased dementia risk in biomarker-positive participants (80% power, two-sided alpha = 0.05). All participants who complete the baseline assessment will be invited to a follow-up evaluation after a minimum of two years. In addition, linkage to Swedish national health registers will be used to ascertain subsequent dementia diagnosis. For the pilot study described here, no formal sample size calculation was performed. Recruitment continued until the study procedures had been adequately tested and participant recruitment and data collection workflow could be smoothly conducted by the study nurse, laboratory personnel and participants.

### Data collection instruments

#### Screening question for memory complaint

Subjective memory complaints were assessed using a single question: “Do you feel like your memory is becoming worse?”. Response options included “no”, “yes, but this does not worry me” and “yes, this worries me”. Both “yes” options fulfilled the criteria of having memory complaints and to be included in the study. The question has been validated as an effective instrument to identify individuals with subjective memory impairment in the MOPEAD study [30, 31].

#### Baseline demographic questionnaire

A structured questionnaire was developed to collect data on memory complaint duration, family history of dementia, education, smoking, alcohol use, social isolation, physical activity and depression. Depression risk was assessed using the patient health questionnaire-2 (PHQ-2), where a score of 3 or higher indicate depression risk. Body mass index (BMI) was calculated from direct measurements of weight and height at the PHC. Alcohol use was categorized as never, moderate (5–9 drinks per week for women or 10–14 drinks for men), or heavy (above these amounts) [32]. Physical activity was considered adequate if participants engaged in at least 150 min per week of high-intensity (jogging, aerobics or tennis) or moderate-intensity (walking, gardening) exercise [33]. The questionnaire was collected and managed using REDCap (Research Electronic Data Capture) hosted at Karolinska Institutet.

#### Digital cognitive tests

Multidomain cognitive testing was conducted using the Mindmore platform (www.mindmore.com), which provides digitized versions of traditional cognitive tests. The battery assesses five cognitive domains: memory, attention and processing speed, executive functions, visuospatial functions, and language [34, 35]. The battery included the following tests: Symbol Digits Processing Test (SDPT) assessing visual scanning; Rey Auditory Verbal Learning Test (RAVLT), measuring verbal learning and memory; the Stroop colour-word test, assessing inhibitory control and executive function; the Corsi Block-Tapping Test, measuring visual short-term and working memory; the Trail Making Test (TMT) Parts A, B and D, assessing mental speed, cognitive flexibility and motor speed, respectively; the Paced Auditory Serial Addition Test (PASAT), measuring information processing; the Cube Drawing Test, assessing visuoconstructive ability; the FAS verbal fluency test, assessing phonemic fluency; and the 15-item Boston Naming test (BNT). Previous studies have demonstrated acceptable comparability between Mindmore’s digital tests and their traditional paper-based counterparts [34, 35]. Convergent validity with other traditional neuropsychological tests has been reported for a subset of the tests [36]. Test-retest reliability and practice effects have been evaluated for most measures in healthy adults, indicating sufficient stability for repeated assessment [37]. High usability and acceptability have been shown in older adults and clinical post-surgical populations with supervised in-clinic testing [38, 39], as well as for remote, unsupervised test administration in a healthy sample [36]. The platform incorporates normative models, adjusting for age, sex, education and input device (mouse, touchpad or touchscreen) based on performance in the cognitively healthy Swedish-speaking population, enabling automated norm-referenced interpretation of test results [35].

The tests were self-administered using a computer interface, operated with either mouse or touchpad, with standardized written and audio instructions. Responses were captured as clicks, response times, on-screen drawings, and microphone-recorded speech. On-screen responses were scored automatically in real-time, drawings and speech were scored by trained Mindmore personnel using predefined protocols. The tests SDPT, TMT, Stroop, Corsi and PASAT were preceded by practice trials that had to be completed successfully for all except the PASAT test; if a participant failed five practice attempts, the test was discontinued, and no performance score was recorded. Participants who did not complete a given test were excluded from summary statistics for that test.

For this study, the Swedish translation of MMSE Norwegian version was used [40] and was administered digitally at the PHC center by the study nurse who read items aloud and recorded responses on the Mindmore platform. Participants used pen and paper for “Write a sentence” and “Figure copying” tasks. The total score was calculated automatically.

### Blood based biomarkers

Four ml of blood was collected in EDTA tubes at a laboratory facility connected to the PHC center. The blood was stored at room temperature for a maximum of 3 h or in the fridge for a maximum of 24 h. Plasma was prepared by centrifugation at 2000 g for 10 min, followed by transferring the supernatant to a sterile polypropylene cryo tube. The plasma was immediately frozen and stored at − 20 °C for up to a month and then transferred to a lab at Karolinska Institutet for aliquotation and storage at − 80 °C until BBM analysis.

Plasma samples were evaluated for 4 different BBMs using the S-PLEX platform from Mesoscale Discovery (MSD). It is a sandwich immunoassay, in multi-well plate format with electrochemiluminescent detection that allows for the ultrasensitive multiplexed quantification of up to 10 analytes per sample in a single run. The selected BBMs for the pilot study were glial fibrillary acid protein (GFAP) as a marker for astrocytosis, neurofilament light polypeptide (NF-L) and microtubule associated protein Tau (Tau) for neurodegeneration, and pTau217 for amyloid pathology. All samples were assayed in duplicates following the manufacturer’s instructions.

### Procedure and patient journey

**Fig. 2:**
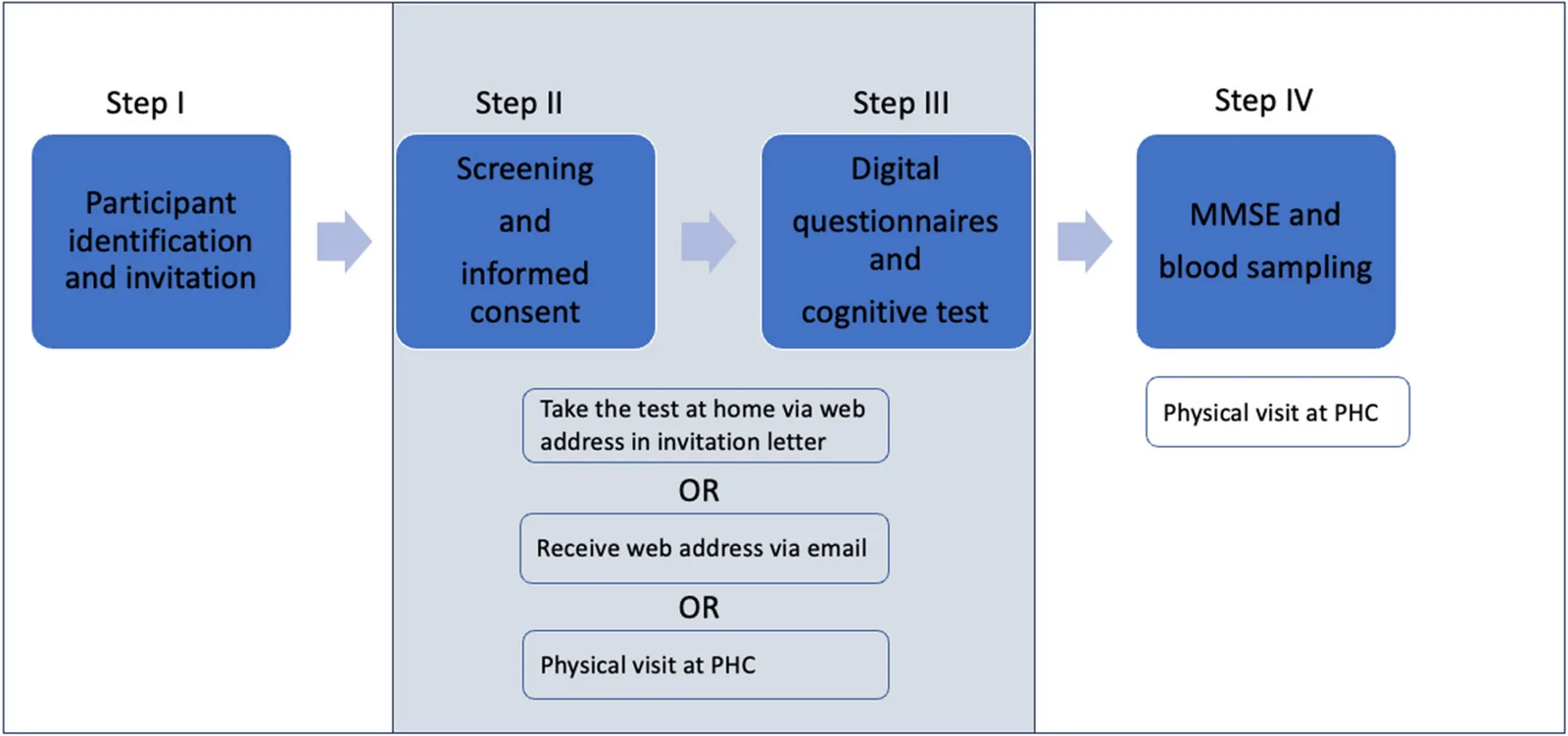
Summary of the participant recruitment steps at baseline. Note. Participants could choose to complete steps II and III at home via a web address provided in the invitation letter or sent via e-mail, or at the primary health care center (PHC). MMSE – mini mental state examination

The participant recruitment consists of 4 steps as depicted in Fig. 2.

#### Step I: participant identification and invitation

Eligible individuals were identified through electronic medical records using MedRave [41]. ICD-10 codes were used to determine inclusion and exclusion criteria. Individuals with a recorded diagnostic ICD code for hypertension (I10) or type 2 diabetes (E11) at any time point were defined as having the condition regardless of the treatment status or other measurements. Medical records were reviewed to exclude individuals who were no longer registered at the PHC, deceased, lacked contact information, or were unlikely to understand the study language, for example, documented need for an interpreter.

Eligible individuals received a postal invitation containing a participant information sheet and a unique web link for study participation. The information sheet described the study purpose, procedures, voluntary nature of participation, right to withdraw at any time, potential risks and benefits, and data privacy procedures. Within 1–2 weeks, a study nurse affiliated with both the PHC center and the research project contacted the participants by telephone to answer questions and assess their interest in participating. Individuals who expressed interest were guided through the next steps, including scheduling a visit at the PHC center. Participants who preferred not to use the web link provided in the invitation letter were instead sent the study web address by email. Individuals who could not be reached by telephone received a text message containing study information and instructions on how to proceed.

#### Step II: screening and informed consent

Screening for subjective memory complaint and collection of informed consent were conducted digitally using REDCap. Participants choosing home-based assessment answered the screening question and provided informed consent through the web link provided in the invitation letter or by email. Participants preferring assessment at the PHC center completed these procedures digitally during their study visit.

#### Step III: digital questionnaire and cognitive testing

After providing informed consent, participants completed a set of demographic questionnaires followed by the digital cognitive test battery at the same session. Home-based testing was unsupervised and conducted on participants’ own computer, with input devices self-reported prior to testing. At the PHC center, testing was conducted on a laptop with a mouse and headphones. Before testing, the study nurse prepared the computer and provided instructions on its use. Participants then completed the questionnaires and cognitive assessment independently in a private study room. The study nurse observed test initiation and remained available, but in a separate room, for technical assistance if required.

#### Step IV: PHC visit

Participants attended the PHC center for measurement of height and weight, MMSE assessment, and blood sampling performed at a laboratory located in the same building. Those who had completed the questionnaires and cognitive testing at home attended the PHC center only for these procedures, whereas participants who choose to do the assessment at the PHC center completed all study procedures during a single visit.

At the PHC, participants were guided by the study nurse wearing the standard uniform used in routine clinical practice to a private room designated for the study. The room was used by one participant at a time to ensure privacy during testing. The PHC visit was conducted solely for research purposes. Individual test results were not shared with treating physicians or participants and were not used for clinical decision-making. Participants who expressed concerns about their memory were advised to consult their physician through the usual clinical care pathway.

### Statistical analysis

To determine feasibility, we assessed parameters related to participant recruitment and data collection, including the numbers of eligible participants identified, invited, and agreeing to participate, the number providing informed consent and included, the proportion completing the digital cognitive test either at home or at a PHC. We also examined whether the study design successfully reached the intended population, i.e., individuals at risk of cognitive impairment. Participant characteristics and cognitive test results were summarised using descriptive statistics, with means and standard deviations (SDs), median and interquartile range (IQR) for continuous variables and percentage for categorical variables. Digital cognitive tests were reported as raw scores (mean and SD) and standardised z-scores adjusted for age, sex, education level and input device. The z scores were categorised as normal (> -1 SD), 1 SD below (≤ -1 SD), 2 SD below (≤ -2 SD) and 3 SD below (≤ -3 SD) the normative score. For the cube test, responses were categorised as correct or incorrect. For BBMs, calibration plots were used to determine equivalent cutoff values of pTau217 between detection methods. Distribution plots were used to show the percentage of samples with relative pTau217 levels and raw biomarker levels. Violin plots were used to show the distribution of biomarker levels across risk and age groups. Data analyses were performed using R version 4.4.2.

## Results

### Recruitment and enrolment process

**Fig. 3:**
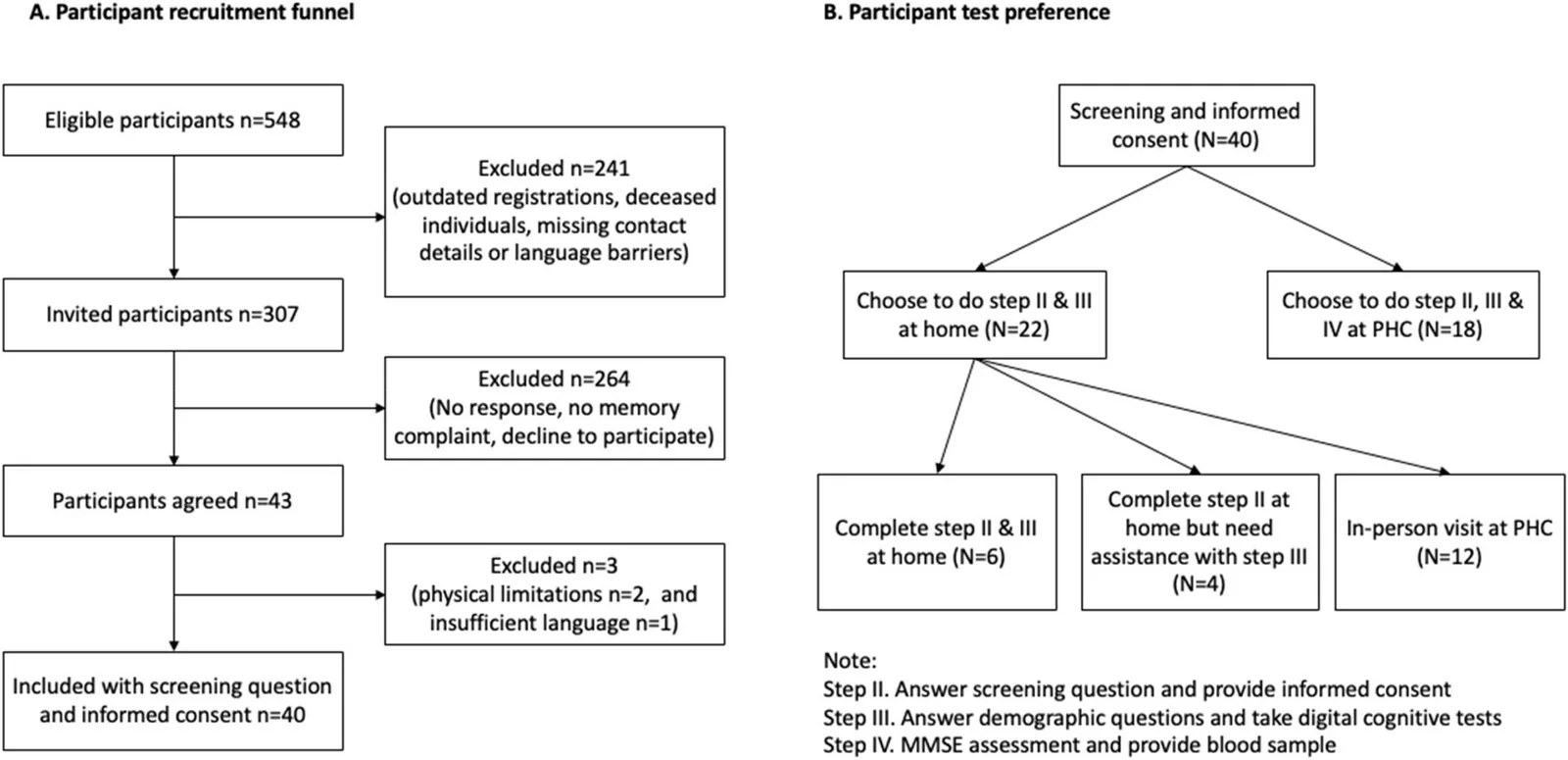
Participant recruitment and cognitive testing preferences in the pilot study. **A**. Participant recruitment funnel illustrating the step-by-step recruitment process. **B**. Participants' preferred cognitive testing modalities and the modalities actually completed

The participant recruitment funnel for the pilot study is shown in Fig. 3A. Of 548 potential participants identified through electronic medical records, 307 were invited by letter. Of these, 43 agreed to participate, fulfilled the inclusion criteria of subjective memory complaint, and provided informed consent, but three were excluded due to physical limitations (*n* = 2) and insufficient language comprehension (*n* = 1) resulting in 40 enrolled participants. This corresponds to 13% participation rate. Subgroup distribution among invited participants included 84 individuals without hypertension or type 2 diabetes (group a), 75 with hypertension only (group b) and 148 with type 2 diabetes, with or without hypertension (group c). Participation rates were similar across the risk groups: 10% in group a, 13% in group b and 14% in group c.

### Data collection process

None of the participants used the web link provided in the invitation letter, therefore it was removed after 50 invitations. Of the 40 enrolled participants, 22 initially opted to complete Steps II and III at home via a link sent by e-mail, while 18 chose to complete all steps at the PHC. However, only six of the 22 completed both steps at home, four completed Step II and part of Step III before requiring nurse assistance, and 12 ultimately completed all steps at the PHC (Fig. 3B).

Three participants did not complete all components of the data collection process: for one participant, cognitive test data were not saved; for another participant, MMSE data was not entered into the digital assessment platform; and one participant did not provide a blood sample. Thus, in total, 37 participants completed all study components.

### Participant characteristics

Baseline characteristics of the 40 enrolled participants are summarised in Table 1. The participants age ranged from 66 to 86 years, with a mean age of 71.6 years. The gender distribution was relatively balanced, with 42% men and 58% women. The education attainment was high with 48% who attended collage/university, 37% who completed secondary education and 15% who completed primary education. Most participants (68%) reported Swedish as their native language. MMSE scores ranged from 23 to 30, with a mean score of 27.7.

**Table 1:** Baseline characteristics of the study participants (*n* = 40)

Variable	Value
Age (years), mean (SD)	71.6 (5.3)
Female, *n* (%)	23 (58%)
Family history of dementia, *n* (%)	19 (48%)
Education, *n* (%)	
Primary	6 (15%)
Secondary	15 (38%)
Post secondary	19 (48%)
Marital status, *n* (%)	
Never married	3 (8%)
Married/cohabiting	23 (58%)
Divorced/separated	7 (18%)
Widowed	7 (18%)
Mother tongue, *n* (%)	
Swedish	27 (68%)
Other	13 (32%)
Smoking, n (%)	
Never smoked	18 (45%)
Former smoker	18 (45%)
Current smoker	4 (10%)
Smokeless tobacco, *n* (%)	
Never used	30 (75%)
Current user	10 (25%)
Alcohol consumption, *n* (%)	
No drinking	15 (38%)
Moderate drinking	24 (60%)
Heavy drinking	1 (2%)
Physical activity, *n* (%)	
less than recommendation	12 (30%)
meet recommendation	28 (70%)
Social contact, *n* (%)	
Daily	27 (68%)
At least weekly	13 (32%)
Risk of depression, *n* (%)	
No risk (PHS2 < 3)	32 (80%)
Risk of depression (PHS2 > = 3)	8 (20%)
BMI, mean (SD)	26.9 (4.5)
MMSE score, mean (SD)	27.7 (1.7)

*SD* Standard deviation, *PHS2* Patient health questionnaire-2, *BMI* Body mass index, *MMSE* Mini-mental state examination

Table 2 presents the symptoms assessments of the participants. Most participants (52%) were from risk group c (type II diabetes with or without hypertension). 82% reported that their memory complaints did not cause concern. The duration of memory complaints was reported as: less than 6 months (12%), 6 months to 1 year (32%), 1 to 2 years (38%) and more than 2 years (18%), respectively. Additionally, 62% reported that their memory symptoms had not worsened over time.

**Table 2:** Risk group and symptom assessment (*n* = 40)

Variable	Value
Risk group	
a) aged ≥ 65 years without hypertension or type 2 diabetes	8 (20%)
b) aged ≥ 65 years with hypertension but without type 2 diabetes	11 (28%)
c) aged ≥ 65 years with type 2 diabetes, with or without hypertension	21 (52%)
Do you feel like your memory is getting worse?	
Yes, but this does not worry me	33 (82%)
Yes, this worries me	7 (18%)
How long have you been aware of your memory problem?	
less than 6 months	5 (12%)
6 months to 1 year	13 (32%)
1 to 2 years	15 (38%)
more than 2 years	7 (18%)
Does your memory problem get worse overtime?	
No	25 (62%)
Yes	15 (38%)

### Cognitive test results

**Fig. 4:**
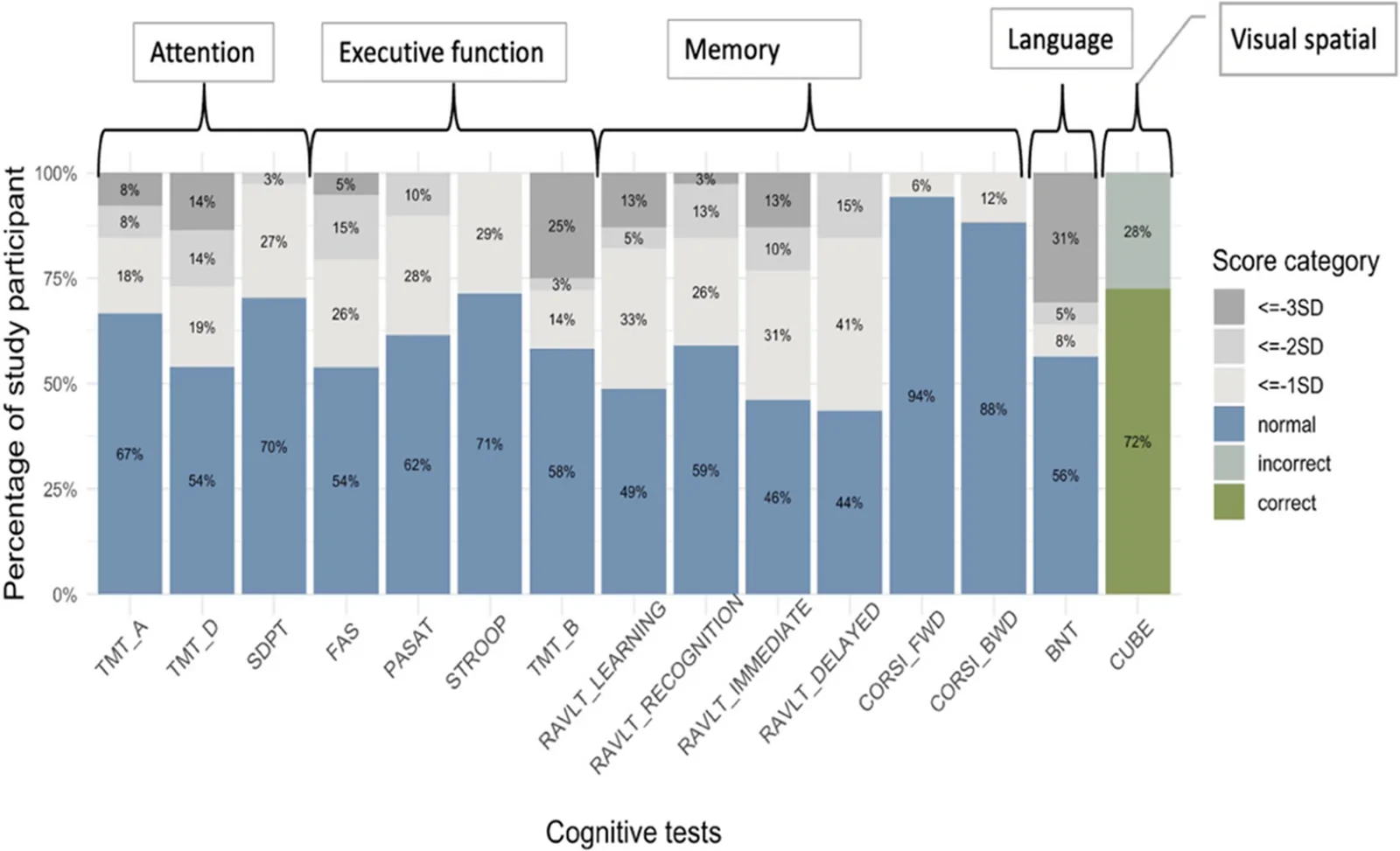
Cognitive test score distribution. Note. The Digital cognitive tests were converted to standardised z-scores adjusted for age, sex, education level and input device. The z scores were categorised as normal (> -1SD), 1 SD below (<= -1SD), 2 SD below (<= -2SD) and 3 SD below (<= -3SD) the normative score. For the cube test, responses were categorised as correct or incorrect

The multidomain digital cognitive test battery was completed by 39 participants in the pilot study. The median time to complete the test battery was 51 min (IQR = 46.5–57.0). The raw scores and standardized z scores were summarized in Table 3. Failures to complete practice trials were observed in one participant in TMT D, two in SDPT, two in TMT B, three in Corsi forward and five in Corsi backward test and hence these tests were excluded from summary statistics in Table 3. Figure 4 shows that approximately one-third of participants scored below the normal range, i.e., performing ≤ − 1SD on each of the cognitive tests administered based on their normative z-scores, adjusted for age, sex, level of education and input device. In addition to effect of age, sex and education, test performance is known to be affected by language-proficiency. The pilot sample included a relatively high proportion of non-native Swedish speakers (32%), who demonstrated significantly lower performance than native Swedish speakers on the verbally mediated tests: RAVLT learning, RAVLT immediate and delayed recall, FAS, BNT and SDPT (Wilcoxon rank-sum test; W values = 87, 82, 72, 63.5, 11, 72.5, all *p* < 0.05). No significant language-related differences were observed for the remaining cognitive tests (all *p* > 0.05).

**Table 3:** Cognitive test scores

Cognitive domain and test results	*n*	Raw score		Z-score	
		Mean (SD)	Min-Max	Mean (SD)	Min-Max
Attention and processing speed					
TMT A time to completion	39	55.4 (19.9)	30.6–116.9	-0.6 (1.4)	-4.3–1.3
TMT D time to completion	37	43.5 (76.2)	20.9–492.5	-3.9 (17.4)	-106.2–1.2
SDPT	37	29.0 (6.2)	14–40	-0.5 (0.9)	-2.8–1.2
Executive functions					
TMT B time to completion	36	95.9 (52.1)	40.2–237	-1.8 (3.3)	-11.2–2.2
PASAT	39	34.5 (13.7)	7–60	-0.5 (1.0)	-2.4–1.5
STROOP index	35	9.3 (2.2)	6–14.6	-0.4 (0.8)	-1.7–1.3
STROOP interference	35	646.0 (402.4)	103–1773	-0.2 (0.8)	-2.1–1.2
Language					
FAS	39	32.1 (13.3)	2–58	-1.0 (1.1)	-3.5–1
BNT	39	9.0 (4.9)	0–15	-1.6 (2.1)	-5.6–1.2
Memory					
RAVLT learning	39	35.3 (14.5)	0–62	-1.1 (1.6)	-5.2–2.3
RAVLT immediate recall	39	6.1 (4.2)	0–15	-1.0 (1.4)	-3.4–2.2
RAVLT delayed recall	39	6.4 (4.0)	0–15	-0.8 (1.3)	-3–1.8
RAVLT recognition	39	9.7 (2.8)	3–15	-0.8 (1.0)	-3.7–1.5
CORSI forward span	36	5.0 (5.0–6.0)*	2–7	0.1 (0.7)	-1.4–1.3
CORSI forward sequences	36	8.0 (6.0–8.0)*	1–12		
CORSI backward span	34	5.0 (4.0–5.0)*	3–7	-0.2 (0.8)	-1.8–1.5
CORSI backward sequences	34	6.0 (5.0–7.0)*	3–10		
Visuospatial functions					
Cube score	39	1.0 (0.0–1.0)*	0–1		
Cube duration	39	108.6 (85.0-175.6)*	33.1–1450.2		

Z-scores are derived by comparison to demographically adjusted norms available on the Mindmore platform

*TMT* Trail Making Test, *SDPT* Symbol Digit Processing Test, *PASAT* Paced Auditory Serial Addition Test, *BNT* Boston Naming Test, *RAVLT* Rey Auditory Verbal Learning Test

*Median (Inter quartile range)

### Biomarkers

The analytical performance for the BBMs using the MSD platform is shown in Table 4. All assays exhibited an excellent analytical performance, with coefficients of variation (CV) under 10% in the calibration curves and recoveries in the range 94–118%. All four BBMs could be determined for all 39 samples. Only four out of 156 determinations (3%) showed a CV over 25%, proving the robustness of the assays.

**Table 4:** Analytical performance of the assays used to quantify blood-based biomarkers

BBM	Standards CV	Recovery (%)	Dynamic range (fg/mL)
GFAP	< 6%	94–109%	1.84x10^2^ - 8.92×10^5^
NF-L	< 10%	96–108%	7.17x10^2^ - 4.53×10^6^
Tau (total)	< 8%	96–107%	9.32 - 1.60×10^5^
pTau217	< 1.5%	94–118%	8.86x10^2^ - 3.63×10^6^

*BBM* Blood based biomarkers, *GFAP* Glial fibrillary acid protein, *NF-L* Neurofilament light polypeptide, *Tau *Microtubule associated protein Tau – Tau, *pTau217 *Tau phosphorylated at 217, *CV *Coefficients of variation

**Fig. 5:**
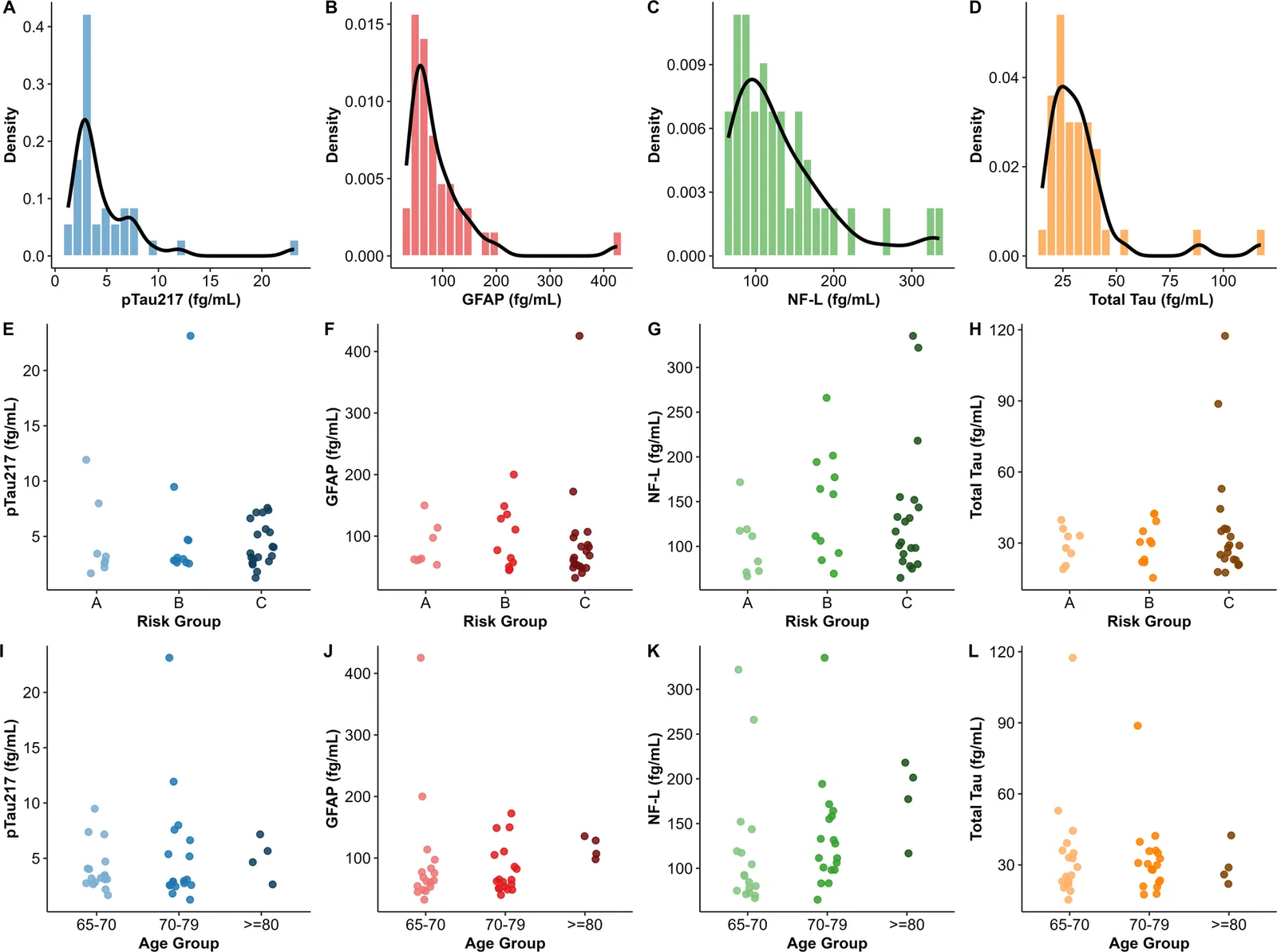
Biomarker distribution.** A**-**D **Distribution histograms showing the frequency of distribution for (**A**) pTau217, (**B**) GFAP, (**C**) NF-L and (**D**) TotalTau. E-H Plots showing distribution of each biomarker stratified by risk groups, A = without hypertension or type 2 diabetes, B = with hypertension only and C = with type 2 diabetes with or without hypertension. (**E**) pTau217, (**F**) GFAP, (**G**) NF-L and (**H**) TotalTau. **I**-**L **Plots showing distribution of each biomarker stratified by age. Individuals are recruited ≥65 years. We show three age categories, between 65-70 years, 70-79 years and >80 years. (**I**) pTau217, (**J**) GFAP, (**K**) NF-L and (**L**) TotalTau. pTau217 = phosphorylated Tau217; MSD S-PLEX = Meso Scale Discovery S-PLEX; GFAP = glial fibrillary acidic protein; NF-L = neurofilament light polypeptide

The biomarkers used in the pilot cohort showed a right skewed distribution (Fig. 5A-D). Moreover, stratification by risk group (Fig. 5E-H) or age (Fig. 5I-L) suggest distinct distributions of biomarkers within these groups. A recent study reported cutoff values for pTau217 - using the Fujirebio platform - to aid AD diagnosis [42]. Here, we compared the MSD pTau217 values with the values obtained from the Fujirebio pTau217 assay in 20 patients clinically diagnosed with AD and 20 age- and sex-matched controls from the Swedish National study on Aging and Care in Nordanstig (SNAC-N) [43]. We performed linear calibration to align MSD values to the Fujirebio scale, showing a strong positive association between the assays (Supplementary Figure S1). The calculated equivalent single cutoff value was 6.60 pg/ml and the double cutoff values were < 5.35 pg/ml and > 8.35 pg/ml for the MSD platform. These equivalent cutoffs were used to estimate the number of samples of this pilot cohort that have low, mid or high pTau217 levels. The single cutoff values provided 9 high and 30 low pTau217 (Table 5), while the double cut-off values provided 4 high, 7 mid and 28 low pTau217 individuals (Table 5).

**Table 5:** Sample count of PREDEM samples stratified as low, mid or high pTau217 based on published cut-off values

	Single cutoff	Double cutoff
Low pTau217	76.9%	71.8%
Mid pTau217	-	17.9%
High pTau217	23.1%	10.3%

## Discussion

**Fig. 6:**
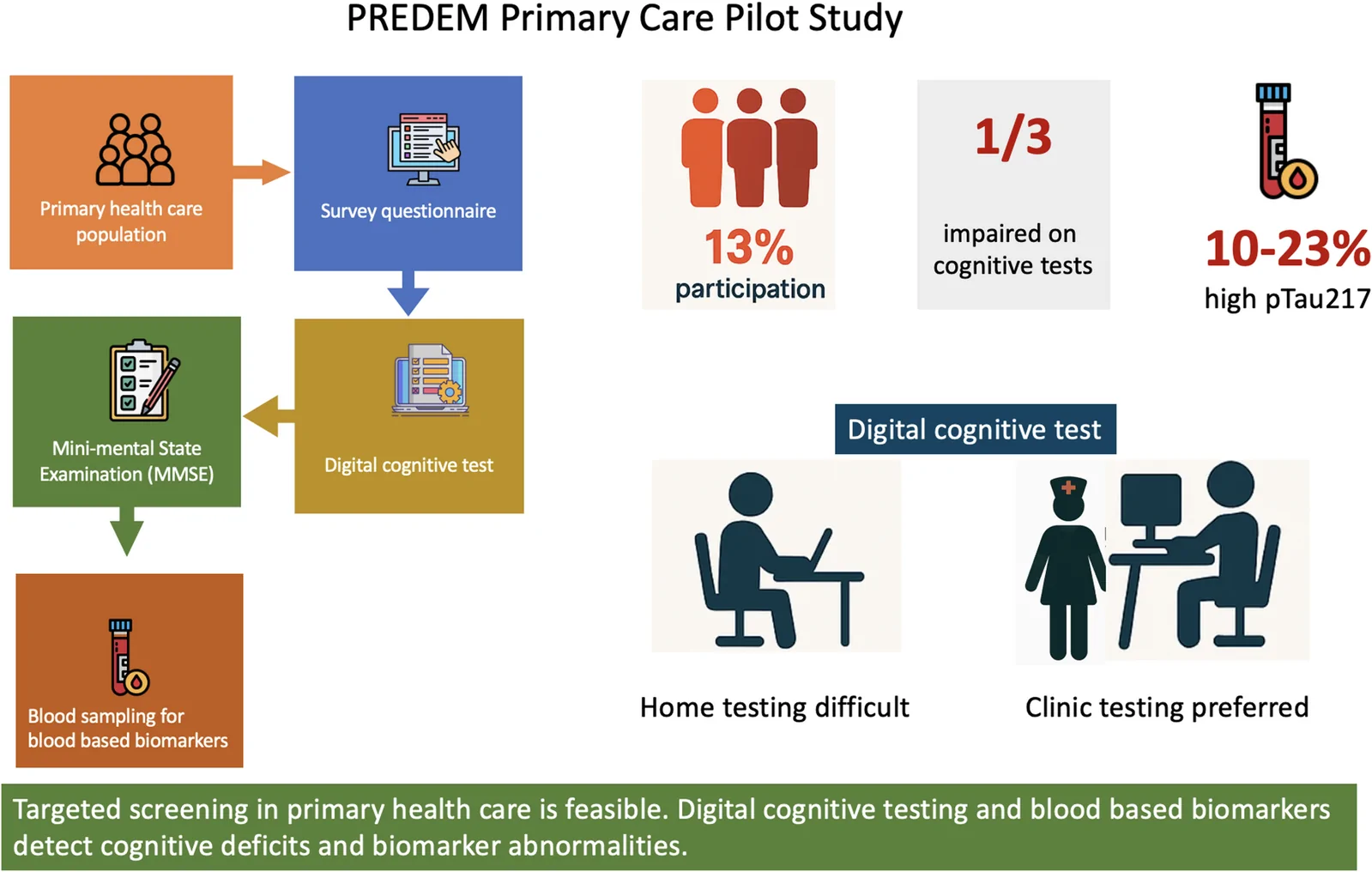
Summary of findings from the pilot study

This pilot study evaluated the feasibility of recruiting older adults at risk of dementia and implementing digital cognitive testing and blood-based biomarkers for PREDEM primary care study. The results are summarized in Fig. 6. The overall participation rate was modest (13%) and the study provided valuable insights for refining the protocol for the full-scale study.

### Recruitment feasibility

Electronic medical record identification using MedRave was efficient but required manual review to address outdated registrations, deceased individuals, missing contact details, and language barriers. The low participation rate may partly be explained by the inclusion criterion requiring self-reported memory complaints, which is a subjective perception that varies widely by age; with 38% in those aged 60–69 to over 60% in those aged over 80 [44]. Additional factors such as anxiety about cognitive testing [45] and fear of learning about own brain health [46] did likely contribute to the low participation. Some invitees perceived memory changes as normal aging, a misconception reported by 79% of individuals in high-income countries [47]. Additionally, individuals with AD may experience agnosia, a lack of awareness of their cognitive deficits. To reduce missed cases due to agnosia, anxiety or fear of learning about brain health, the protocol has been revised for remaining recruitments to include all eligible individuals regardless of subjective complaints. We retain the screening question to understand whether those with and those without subjective memory complaint have different risks of dementia development in the main study.

### Digital screening, questionnaire and cognitive testing - preferences and barriers

None of the participants used the web link provided in the invitation letter to complete the screening questions; instead, most preferred receiving email or proceeding directly to in-person screening. Although 55% of participants expressed interest in home-based digital testing, which is consistent with previous findings [48], only 15% completed the demographics questionnaire and the cognitive test battery at home without assistance. This discrepancy highlights a gap between stated preferences and practical feasibility in this population.

Barriers to home-based testing included technical requirements (access to a computer with microphone, speakers and a compatible browser) as well as the need to complete multiple steps within a single session. To address these challenges to the testing context and ensure standardized administration, the protocol was adapted so that all steps will be conducted at PHC centers under nurse supervision, which proved feasible in this study. Although digital testing is initiated under supervision, subsequent tasks are performed unsupervised. Thus, this set-up remains a time-efficient approach for collecting rich data on cognition.

### Cognitive battery and participant burden

The study employed a comprehensive cognitive test battery, with a median completion time of 51 min, which may be too lengthy for a PHC setting. However, the choice of comprehensive battery is intentional, as it will enable evaluation of the diagnostic contribution, feasibility, and completion time of individual subtests. These data will help identify an optimal, shorter combination of cognitive tests and biomarkers for future primary care use and may also provide insights into which cognitive measures best differentiate AD from dementia due to other etiologies.

Based on feedback from the pilot study, the PASAT test was removed because it was perceived as demanding and stressful for participants. The clock-drawing test was added because it is brief and commonly used in routine clinical practice. These modifications were intended to improve participant acceptability and enhance the clinical relevance of the cognitive assessment.

Some single tests were omitted for participants who failed to complete practice trials. Unsuccessful practice completion may reflect difficulties with task comprehension or underlying cognitive limitations. Consequently, the proportion of participants showing cognitive impairment based on completed tests may underestimate the overall level of cognitive difficulty in the sample. In this study, cognitive impairment was defined operationally as performing at least 1 SD below normative data on one or more cognitive tests. Although this definition does not correspond to a clinical diagnosis of mild cognitive impairment [17, 49], it was chosen to facilitate interpretation of cognitive performance in this pilot cohort. Notably, despite the potential underestimation resulting from incomplete test data, more than one-third of participants met this criterion, suggesting that the risk-targeted recruitment strategy successfully identified individuals with objective cognitive deficits.

### Language and test validity

Language proficiency significantly affected performance on verbally mediated tests (RAVLT, FAS, BNT), with non-native Swedish speakers scoring lower on these tests. This aligns with previous evidence that language barriers can lead to misinterpretation of test results and false indications of cognitive impairment [50, 51]. For bilingual individuals, cognitive testing should consider language skills, cultural background, and test adaptation [51]. In the present study, cognitive testing and normative interpretation are based exclusively on Swedish-language versions of the tests and Swedish normative data. Accordingly, inclusion criteria were refined to ensure sufficient Swedish language proficiency in the full-scale study.

### Blood-based biomarkers

BBM analysis demonstrated the validity of identifying BBMs that can be used to identify individuals at risk of developing cognitive impairment and AD. One limitation of this pilot study is the lack of statistical power to compare biomarkers across risk groups and ages due to the small sample size. This problem will be resolved in the target cohort of 1200 individuals. Despite this lack of statistical analysis, we observed some trends in altered biomarker levels across ages and the three risk groups. The right skewed distributions of pTau217, GFAP, NF-L and total Tau suggest that we have identified individuals with abnormal levels of these biomarkers, supporting their relevance in primary care. This notion is supported by the high levels of pTau217 in 23% or 10% of the participants, using single or double cutoffs, respectively. pTau217 is good at predicting amyloid pathology in individuals that display some cognitive symptoms [42]. However, the positive predictive value of pTau217 for future dementia is low in the general population [23]. One limitation with the other biomarkers, GFAP, NF-L and total Tau, is that they are elevated in neurodegenerative disorders in general, and thus not specific for AD. Hence, we will in the large-scale study expand the biomarker toolbox to include additional biomarkers, for instance glycans, which have shown remarkable ability to predict future cognitive decline and AD dementia in recent studies [43, 52, 53]. In the full-scale study, we will investigate associations between cognitive test data and BBM analyses and define the optimal combination of digital cognitive tests and biomarkers for realistic and cost-effective approaches to predict and detect AD in primary care.

### Protocol adaptation

Feasibility studies play a critical role in identifying potential operational challenges and informing protocol refinements before large-scale implementation [54]. In this pilot, several adaptations were made based on observed barriers. These included removing the requirement for subjective memory complaints to reduce selection bias and increased participation rate, shifting all cognitive testing to PHC centers to overcome technical difficulties with home-based assessments. In addition, inclusion criteria were refined to ensure adequate language proficiency, and cognitive battery was refined to reduce participant burden. These changes aim to improve recruitment efficiency, data quality, and participant experience in the full-scale PREDEM-primary care study.

### Targeted screening approach

The PREDEM-primary care study employs a targeted screening approach focusing on individuals aged ≥ 65 years with hypertension and/or type 2 diabetes, complemented by assessment of subjective memory complaint. This approach is based on evidence that hypertension and type 2 diabetes are among the most common diagnoses encountered in PHC [55, 56], have relatively high diagnostic validity compared with many other cardiovascular conditions [57], and are well-established risk factors for AD and related dementia [58]. To minimize inclusion of participants with non-AD cognitive impairment, individuals with major vascular diseases (which are associated with vascular dementia), neurodegenerative disorders, or substance abuse were excluded. In addition, participants are characterized with respect to a broader set of modifiable risk factors including obesity, smoking, alcohol intake and social isolation, enabling a comprehensive assessment of individual risk profiles [58].

Compared to universal screening, which is not recommended due to insufficient evidence and the potential to increase PHC workload [24, 25], this targeted approach may improve resource allocation by prioritizing individuals with elevated risk profiles. Furthermore, this strategy complements existing Swedish studies, such as the BioFinder Primary Care [22] which recruits individuals presenting with memory complaints, and the REAL-AD study which employs universal screening [59].

Although the primary objective of PREDEM is to evaluate the combined diagnostic value of digital cognitive test and BBMs for AD diagnosis, the study design also enables investigation of cognitive decline from other causes. The digital cognitive battery assesses multiple cognitive domains, while the BBMs included both pTau217, a marker of amyloid pathology, as well as GFAP and NF-L, which reflect broader neurodegenerative and neuronal injury processes rather than AD pathology alone. Furthermore, all participants will be followed for two years, and study data will be linked to Swedish health registries to ascertain subsequent clinical diagnoses. The combination of these features will allow assessment of dementia risk and diagnostic outcomes in an at-risk PHC population.

### Implementation in primary care

The present pilot represents an initial step toward the longer-term goal of developing a clinically feasible and interpretable strategy for dementia risk detection in primary care. Pilot findings demonstrate that the targeted screening approach can identify individuals with measurable cognitive deficits and elevated BBMs, supporting the feasibility of participant recruitment. However, the optimal population for targeted testing remains uncertain.

The ongoing PREDEM primary care study will continue recruitment to the planned sample size of 1200 and evaluate how cognitive and BBM test results relate to risk factor profiles and subjective memory complaints. This will provide evidence on the characteristics of the population reached by risk-based screening, their cognitive and biomarker profiles, and which subgroups are most likely to benefit from targeted assessment. In parallel, the study will assess the diagnostic performance, clinical utility, and cost-effectiveness of individual cognitive tests and BBMs.

Successful implementation of this approach in routine primary care will depend not only on the diagnostic performance of the tests but also on the feasibility of administrating the assessments and interpreting results in clinical practice. This is particularly relevant given that the current cognitive test battery required a median completion time of 51 min and administration by a trained research nurse, making widespread implementation unlikely. Data from the main study will therefore be used to identify a shorter and more practical assessment battery and to establish evidence-based thresholds for test interpretation. In addition, the digital cognitive platform provides standardized scoring and visualization of performance relative to normative data, which may facilitate interpretation by primary care providers. Whether these outputs, together with BBM results, improve clinical decision-making and provider confidence and provider acceptability will need to be evaluated in future implementation studies.

## Limitations

This pilot study has several limitations that should be considered. First, the modest participation rate and the requirement for self-reported memory complaints may have introduced selection bias, potentially favoring individuals who are more aware of or concerned about cognitive decline. Participant characteristics, including relatively high education levels, family history of dementia, and levels of physical activity, further suggest a non-representative sample. Second, the small sample size limits statistical power, particularly for subgroup comparisons and detailed analyses of cognitive tests and biomarker distributions across risk groups and age categories. As a result, observed trends in cognitive performance and biomarker levels should be interpreted with caution. Finally, the reliance on Swedish-language cognitive tests and normative data may have affected test validity for non-native speakers, despite efforts to address language proficiency. This may have contributed to lower performance in verbally mediated tasks and limits generalizability to more diverse populations. Future studies should incorporate more inclusive recruitment strategies and validated multilingual tools to improve representativeness and generalizability.

## Conclusion

Our findings indicate that risk-targeted screening of individuals aged ≥ 65 years with memory complaints and comorbid hypertension or type 2 diabetes effectively identifies a relevant population for the PREDEM-primary care study, with approximately one-third scoring below the normal range on each administered cognitive test. Based on the findings in this pilot study, adjustments to the recruitment process and test battery have already been implemented to improve accessibility. The ongoing full-scale study will further evaluate the performance and potential utility of this approach, including its ability to inform future screening strategies in primary health care.

## Supplementary Information

Supplementary Material 1.

## Data Availability

The datasets generated during and/or analysed in the current study are not publicly available due to the involvement of patient information but are available from the corresponding author upon reasonable request.

## Abbreviations

AD: Alzheimer´s disease
Aβ: Amyloid β-peptide
BBMs: Blood-based biomarkers
BMI: Body mass index
BNT: Boston Naming Test
GFAP: Glial fibrillary acid prote
IQR: Interquartile range
MMSE: Mini-Mental State Examination
MoCA: Montreal Cognitive Assessment
MSD: Mesoscale Discovery
NF-L: Neurofilament light polypeptide
PASAT: Paced Auditory Serial Addition Test
PHQ-2: Patient health questionnaire-2
PHC: Primary health care
Tau: Protein Tau
REDCap: Research Electronic Data Capture
RAVLT: Rey Auditory Verbal Learning Test
SDs: Standard deviations
SDPT: Symbol Digits Processing Test
pTau217: Tau phosphorylated at position 217
TMT: Trail Making Test
