# Supplementary material for: Enhancing Alzheimer’s disease diagnosis in primary care through targeted screening of people at risk of developing dementia – study design and feasibility assessment of the PREDEM primary care study
Source: Alzheimers Res Ther. 2026 Sep 14;18:201. doi: 10.1186/s13195-026-02162-w (PMC13576326; doi:10.1186/s13195-026-02162-w)
Supplement: Supplementary file 1 [file 13195_2026_2162_MOESM1_ESM.docx]

**Supplementary Figure 1. Equivalent pTau217 cutoffs using MSD S-PLEX.**

***Note.*** A subset of samples from the SNAC-N cohort with clinical diagnosis of AD or healthy controls (https://doi-org.proxy.kib.ki.se/10.1016/j.ebiom.2025.105598) were run on both Lumipulse (Fujirebio) and MSD S-PLEX platforms to establish equivalent cutoff values. **A** Deming scatter plot comparing MSD S-PLEX pTau217 measurements to Fujirebio pTau217 measurements. **B** Bland-Altman plot comparing both techniques. **C** Equivalent cutoff values of pTau217 using MSD S-PLEX as compared to the Fujirebio platform, for which cutoff values have been published (1). **D** Fraction of PREDEM samples stratified as high, mid, or low pTau217 based on single or double cut-off values. pTau217 = phosphorylated Tau217; MSD S-PLEX = Meso Scale Discovery S-PLEX.

References

1.	Palmqvist S, Warmenhoven N, Anastasi F, Pilotto A, Janelidze S, Tideman P, et al. Plasma phospho-tau217 for Alzheimer’s disease diagnosis in primary and secondary care using a fully automated platform. Nat Med [Internet]. 2025 Jun 1 [cited 2025 Nov 28];31(6):2036–43.
